# Multilayered Porous Titanium-Based 3rd Generation Biomaterial Designed for Endosseous Implants

**DOI:** 10.3390/ma14071727

**Published:** 2021-03-31

**Authors:** George Calin Dindelegan, Alexandra Caziuc, Ioana Brie, Olga Soritau, Maximilian George Dindelegan, Vasile Bintintan, Violeta Pascalau, Carmen Mihu, Catalin Popa

**Affiliations:** 1 Surgical Department, University of Medicine and Pharmacy “Iuliu Hatieganu”, 400349 Cluj-Napoca, Romania; george.dindelegan@umfcluj.ro (G.C.D.); vasile.bintintan@umfcluj.ro (V.B.); 2 Radiobiology and Tumor Biology Department, Oncologic Institute Ion Chiricuta, 400015 Cluj-Napoca, Romania; brie.ioana@iocn.ro (I.B.); soritau@iocn.ro (O.S.); 3 ENT Department, University of Medicine and Pharmacy “Iuliu Hatieganu”, 400349 Cluj-Napoca, Romania; dindelegan.maximilian.george@elearn.umfcluj.ro; 4 Department of Science and Technology, Faculty of Materials and Environmental Engineering, Technical University of Cluj-Napoca, 400114 Cluj-Napoca, Romania; violeta.pascalau@stm.utcluj.ro (V.P.); catalin.popa@stm.utcluj.ro (C.P.); 5 Histology Department, University of Medicine and Pharmacy “Iuliu Hatieganu”, 400349 Cluj-Napoca, Romania; carmenmihu@umfcluj.ro

**Keywords:** biomaterial, microspheres, growth factors, titanium, IGF1, BMP2

## Abstract

This work proposes a novel complex multi-layered material consisting of porous titanium as a substrate and a complex coating consisting of a chitosan film engulfing microsphere loaded with growth factors such as BMP2 (bone morphogenic protein 2) and IGF1 (insulin-like growth factor-1). The microspheres were obtained through deposition of dual layers of calcium cross linked pectin–chitosan/pectin polyelectrolyte onto a BSA (bovine serum albumin) gel core. The multilayer was conceived to behave like a 3rd generation biomaterial, by slow delivery of viable growth factors around implants, and to assist the healing of implantation wound and the development of new vital bone. The biologic effect of the delivery of growth factors was studied in vitro, on MSC-CD1 mesenchymal stem cells, and in vivo, on CD1 mice. Proliferation and differentiation of cells were accelerated by growth factors, especially IGF1 for proliferation and BMP2 for differentiation. In vivo tests analyzed histologically and by MicroCT show a more structured tissue around BMP2 samples. The present concept will give the best clinical results if both growth factors are delivered together by a coating film that contains a double population of microcarriers.

## 1. Introduction

The evolution of in-depth thinking in the field of biomaterials has led in time from a first generation of quasi-bioinert materials, to a second one that exhibits designed chemical reactions in tissue, such as bioactive and bioresorbable materials and, lately, to a third generation of biomaterials conceived to help body heal itself [1].

Titanium, as a first-generation medical material, i.e., quasi-bioinert, is the most frequently used metal for endosseous implants. Nevertheless, titanium-based implants failure is not uncommon, both with regard to the bone-implant interface and as a result of bulk mechanical properties. A number of 722 articles published between 2016 and 2018 refer to toxic effects induced by titanium implants [2]. There is also a direct relation between the risk of bone atrophy around titanium implants and the “stress shielding” effect as a result of the tissue-implant mechanical mismatch [3]. As the Young’s modulus for cortical bone is 7–30 GPa and that of c.p. (commercially pure) titanium or titanium alloys usually employed for implants is more than 110 GPa, mechanical mismatch is, although smaller than in the case of stellites or stainless steel, of major importance. The use of low modulus beta titanium alloys is a way of decreasing the bone atrophy risk [4,5]. This approach is limited by the lowest value of Young’s modulus for polycrystalline beta titanium alloys, of 55 GPa. Thus, for a further increase of the effectiveness of osseointegrated titanium implants, porous structures are to be considered, as in the case of the present paper. More, porous titanium implants are found to prove enhanced osteoconductivity in the early stages of bone healing [6], reducing the effective osseointegration time and leading to a decreased risk of early post-operative failures in the case of uncemented implants [7].

Growth factors (GFs) are generally defined as secreted biologically active molecules that affect the growth of cells [8]. Bone contains different types of growth factors [9], with a major influence upon osteoblast behavior, thus these are being consistently used for bone tissue regeneration [10] and tissue engineering applications [11]. As FDA (Food and Drug Administration) approved for clinical use GFs, bone morphogenic proteins (bone morphogenetic protein 2—BMP2 and bone morphogenetic protein 7—BMP7) mediate the differentiation of mesenchymal stem cells into cartilage/bone forming cells and are essential for new bone formation and bone healing [12]. For BMP2 (bone morphogenetic protein 2), it has been demonstrated to potently induce osteoblast differentiation in a variety of cell types. As an adjuvant to allograft bone or as a replacement for harvested autograft, BMP appears to improve fusion rates after spinal arthrodesis in both animal models and humans, while reducing the donor-site morbidity previously associated with such procedures [13]. However recent studies showed problems like ectopic growth, lesser protein delivery, and inactivation of the protein. This signifies that it is necessary to modify the available carrier systems as well as explore other biomaterials with desired properties [14,15].

Insulin-like growth factors (IGFs) are involved in the regulation of tissue growth and their lack can lead to a delayed wound healing [16]. On purpose delivered insulin-like growth factor-1 (IGF1) is well acknowledged for promoting wound healing in many types of tissue [17,18]. For this reason, on-site delivery of IGF1 was successfully employed for stimulating collagen synthesis in tissues [19]. Insulin like growth factor (IGF-1) particularly influences bone remodelling, a process that involves the removal of mineralized bone followed by the formation of bone matrix by osteoclasts and osteoblasts [20]. Several studies have demonstrated an important role of IGF-1 in angiogenesis [21].

In this paper, we propose a novel porous titanium-based multi-layered material acting as a third-generation biomaterial designed for endosseous implants. The concept starts with porous c.p. titanium compacts obtained via powder metallurgy route using a water-soluble space holder, in view of obtaining of an appropriate porosity in terms of pores size and shape. The active surface of the compacts will be coated with a hydrogel membrane that contains core-shell microcarriers engineered to deliver BMP2 and IGF1 growth factors with the purpose to sustain the development of newly formed bone and to heal the wound in the implantation site. The microcarriers are conceived to protect GFs against proteolytic degradation and are BSA gel-cored, with a dual multi-layer shell, consisting of pectin hydrogel and pectin/chitosan polyelectrolyte [22]. In order to sustain microcarriers and to provide a prolonged delivery of GFs, these are entrapped into a chitosan hydrogel film that is coated on the surface of titanium compacts.

The characterization of the synthesized samples was conducted with the aim of assessing both the structural and bio-functional aspects, in view of recommending the novel concept for further implant development. A great emphasis was given to the biocompatibility of the cellular support structures (coated titanium samples) by evaluating the local and systemic response, the differentiation of stem cells to the bone lineage, and the changes around the implant.

## 2. Materials and Methods

### 2.1. Preparation of Porous Titanium Samples

For the synthesis of porous titanium, we used the following components: commercially pure (c.p.) titanium powder (Ningjin Henfa Ti Powder Factory, Ningjin, China) obtained through the hydride-dehydride process having a particle size smaller than 45 μm and dextrin (Carl Roth GmbH, Karlsruhe, Germany) as space holder, with particle size below 100 μm. Porous titanium samples were made from a mixture of Ti and 35% (%vol.) dextrin, by direct pressing in a 6 mm diameter rigid die (custom), with 400 MPa. Dextrin was removed from green samples by solving in 90 °C hot stirred water for 15 min. The compacts were further dried in an oven (Caloris, Bucharest, Romania) at 90 °C for 30 min. Sintering was performed in vacuum (Leybold-Heraeus, Terryville, CT, USA) (10^−5^) at 1300 °C for one hour, with a heating rate of 5 °C/min. The resulted compacts were studied from the point of view of pore structure by SEM (Jeol-JSM 5600 LV, Jeol, Tokyo, Japan) and optical microscopy (Olympus GX 51, Tokyo, Japan).

### 2.2. Cells Harvesting and Cultivation

All experiments were performed in accordance with the current legislation on animal rights, with approval of the Ethics Committee No. 434/20.07.2015.

Adult mesenchymal cells (MSCs) were isolated from the bone marrow (BM) of CD1 mice, applying the ethical standards imposed by the European Union, (Directive 2010/63/EU of the European Parliament and of the Council of 22 September 2010 on the protection of animals used for scientific purposes).

The medullary canal was washed using complete Dulbecco’s Modified Eagle’s Medium (DMEM) with 10% Fetal Bovine Serum (FBS), 1% Penicillin/Streptomicin solution, 1% Glutamine and 1% Non-Essential Amino acids (NEA). The cells harvested from the medullary canal were washed, passed through a sterile Filcons filter (BD Biosciences, San Jose, CA, USA), with a 70 μm network), and centrifuged (Hettich Universal 32R, Andreas Hettich GmbH&Co, Tuttlingen, Germany), at 4 °C, 1000 rpm, 5 min) to a final concentration of 5 × 10^6^ viable cells/mL. They were seeded on 25 cm^2^ cultureflasks (Nunc^TM^, ThermoFisher Scientific, Waltham, MA, USA) and incubated at 37 °C in atmosphere enriched with 5% CO_2_ and 95% humidity. The culture medium consisted of: DMEM with 4.5 g glucose/liter in combination with F-12 HAM medium (ratio 1:1), 20% FBS, 100 U/mlPenicillin, 100 μg/mL Streptomicin, 2 mM L-glutamine, 1% NEA, 55 mM beta-mercaptoethanol, and 1 mM natrium piruvate. All reagents were purchased from Sigma-Aldrich, St. Louis, MO, USA. When the cells became confluent, they were re-plated by trypsinization (trypsin-EDTA, Sigma-Aldrich, St. Louis, MO, USA), at a 1:3 split ratio, and recultured until an adequate number of cells was obtained. At the 6th passage, when the cultures presented unitary fibroblastoid-like morphological characters and high proliferative potential, the cells were characterized by immunocytochemistry for stemness markers expression: Octamer binding transcription factor (Oct3/4, Santa Cruz Biotechnology, Dallas, TX, USA), SRY-Box Transcription Factor 2 (Sox-2, Santa Cruz Biotechnology, Dallas, TX, USA), Stage-Specific Embryonic Antigen 1 (SSEA-1, Santa Cruz Biotechnology, Dallas, TX, USA) and Nanog (Santa Cruz Biotechnology, Dallas, TX, USA). The isolated cells were positive for all these markers (data not shown in this paper).

### 2.3. Preparation of the Growth Factors Releasing Titanium-Based Samples

In the in vitro experiments, titanium samples were used as follows: three types of porous titanium samples were employed: (a) simple, uncoated, as controls; (b) coated samples, containing IGF1; (c) coated samples, containing BMP2.

To study the influence of the growth factors, the implants were coated with chitosan layers (Sigma Aldrich, Saint Louis, MO, USA) containing microcapsules (3 × 10^6^/implant) loaded with BMP2 and IGF1 respectively. After sterilization with ethylene oxide (Sigma Aldrich, Saint Louis, MO, USA) to avoid titanium denaturation and the inactivation of growth factors), the samples were placed in 96-well plates (Nunc^TM^, ThermoFisher Scientific, Waltham, MA, USA) with culture medium containing the cells (5 × 10^4^ cells/implant).

### 2.4. The Enzyme-Linked Immunosorbent Assay (ELISA)

The samples were then collected from the wells at different intervals (day 2, 3, 7, 10, 14, 17, 21). They were evaluated by ELISA using specific kits for the measurement of BMP2 and IGF1 released in the culture medium.

BMP2 and IGF1 levels were determined using specific R&D Quantikine ELISA kits (R&D Systems, Minneapolis, MN, USA), according to the manufacturer’s instructions. Briefly, standards and samples were added in the wells, incubated for 2 h on a shaker (Titramax 1000, Heidolph Instruments GmbH&Co, Schwabach, Germany) at room temperature and washed to remove the unbound protein. Then, specific conjugates were added, followed by incubation and further washing. After the final 30 min incubation with the substrate solutions, the stop solutions from the kits (Quantikine, R&D Systems, Minneapolis, MN, USA) were added and optical densities were determined with a microplate Biotek Synergy2 reader (Synergy HT; BioTek, Winooski, VT, USA) set to 450 nm. We used duplicates for each sample. The optical densities and concentration values were derived from standard curve.

### 2.5. Viability and Proliferation Assay

BM MSCs were seeded onto the titanium implants after staining with the lipophilic membrane dye PKH26 Red Fluorescent Cell Linker Kit (Sigma-Aldrich, Saint Louis, MO, USA). 1 × 10^6^ cells were washed twice with Phosphate Buffered Saline (PBS, Sigma-Aldrich, St. Louis, MO, USA) by centrifugation at 1000 rpm for 5 min and suspended in 1 mL staining solution containing 4 µL PKH26/mL. The PKH26 staining was stopped after 5 min by adding 10 mL culture medium containing 10% FBS. The cells were counted and 5 × 10^4^ cells were seeded onto each titanium disc placed in 96 well plates in standard stem cell medium. Triplicates were done for: porous titanium, Ti-BMP2 (porous titanium-chitosan film-microcapsules system + BMP2), Ti-IGF1 (porous titanium-chitosan film-microcapsules system + IGF1), and samples with cells cultivated on plastic surface (Nunc^TM^, ThermoFisher Scientific, Waltham, MA, USA) as controls. Red fluorescent cells were visualized by fluorescence microscopy with a Zeiss Axiovert D1 microscope (Zeiss GmbH, Jena, Germany), using filters at 546 nm and images were captured after 2 h, 48 h, and 7 days of cultivation.

### 2.6. Differentiation Assay

The differentiation of stem cells (BM MSCs) into bone cells under the influence of growth factors delivered by the titanium-chitosan film-microcapsules system was studied by evaluating the expression of osteogenic markers after 7 and 21 days of cultivation of cells on titanium implants. The culture medium consisted of DMEM high glucose/F-12 HAM medium (1:1 ratio) 10% FBS, 100 U/mL Penicillin, 100 μg/mL Streptomycin, 2 mM L-Glutamine, 1% NEA. Immunocytochemical staining was performed at day 7 for osteopontin (OP) as an early osteogenic marker and for actin F (phalloidin staining) as a marker of cytoskeleton reorganization. After 21 days of cultivation, the expression of osteocalcin (OC) and osteopontin (OP) were determined. The protocol of immunostaining consisted of a fixation step with 4% paraformaldehyde (Fluka, Buchs, Switzerland) followed by permeabilization of cellular membranes with 0.1% Triton-X100 solution (Sigma-Aldrich, St. Louis, MO, USA). Exposure of samples to 10% BSA (bovine serum albumin) (Sigma, St. Louis, MO, USA) for 15 min was used for blocking the non-specific binding of antibodies. The samples were incubated overnight at 4 °C with the primary antibodies OP and OC (Santa Cruz Biotechnologies, Dallas, TX, USA) at a dilution of 1:50. Then the samples were incubated for 45 min with the secondary antibodies conjugated with Fluorescein-5-isothiocyanate (FITC, Santa Cruz Biotechnology, Dallas, TX, USA) and Texas red (Santa Cruz Biotechnologies, Dallas, TX, USA). Phalloidin conjugated with Tetramethylrhodamine (TRITC, Sigma-Aldrich, St. Louis, MO, USA) was added to samples fixed at day 7 and incubated for 20 min. Each step of the staining was followed by 3 washes with PBS. For the visualization of the nuclei, a mounting medium containing 4′,6-diamidino-2-phenylindole (DAPI, Sigma-Aldrich, St. Louis, MO, USA) was added. Titanium implants were then analysed in fluorescence with an inverted microscope Zeiss Axiovert D1 (Zeiss GmbH, Jena, Germany) using filters at 488, 546 and 346 nm. The images were captured with a CCD camera, MRM Axiocam (Zeiss GmbH, Jena, Germany).

### 2.7. Biological Evaluation of the Samples

In order to test the in vivo response and biological effectiveness of the proposed multilayer 3rd generation biomaterial, we used CD1 male mice, aged 16 weeks. All experiments were performed in accordance with current legislation on animal rights, with approval of the Ethics Committee. A group of 20 animals was selected and all animals were implanted subcutaneously on both sides, front/rear, with: a porous Ti control sample (without active coating); a BMP2 releasing sample; an IGF1 releasing sample. The handling of explants was minimal in order to avoid the mobilization of stem cells. The adjacent zones of explants retrieved after 14 days were analyzed either by histology means or by Micro-CT (Skyscan 1172, Bruker, Billerica, MA, USA), in view of assessing the effect on the structure and density of interfacial tissue.

## 3. Results

### 3.1. Evaluation of Ti samples

**Figure 1:**
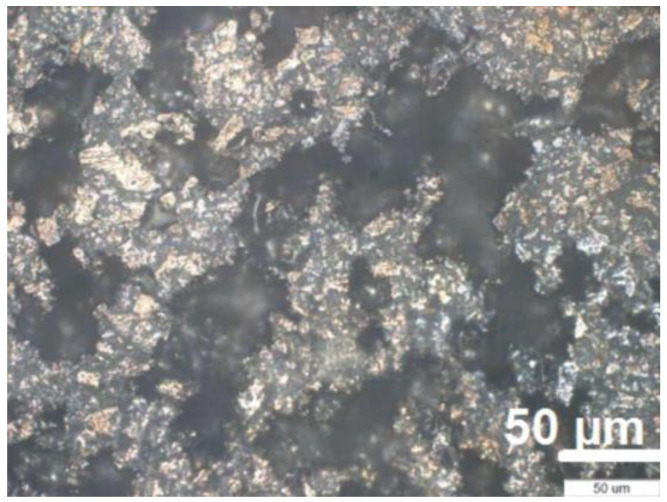
Green c.p. Ti sample, optical microscopy.

After compaction and water solving of the dextrin space holder, the structure of green samples was porous, with interconnected pores, Figure 1.

**Figure 2:**
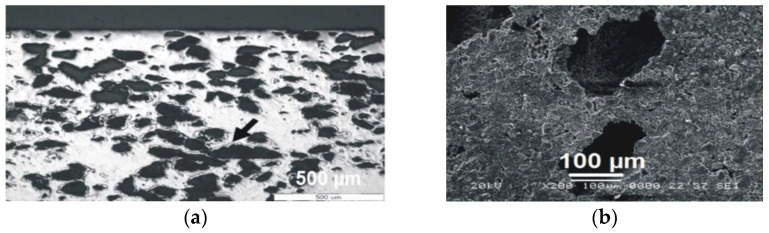
Sintered c.p. Ti sample: (**a**) general aspect (optical microscopy), unetched sample; (**b**) details on the shape of pores (SEM).

After the sintering stage, the samples showed a 36% open porosity, as shown by Arhimede’s method; the pores were found to be appropriate for vital bone ingrowth, both due to their round shape and their size. Several pores were more than 150 μm in diameter, allowing for cellular evolution towards fully evolved osteon units, Figure 2. In the structure of the unetched sample in Figure 2a, a light color phase can be seen close to the pores (dark arrow), showing that residues of dextrin reacted upon sintering and formed complex compounds, which were found not to impede upon biocompatibility, as proven elsewhere [23]. Nevertheless, the final samples were tested first in vitro, to assess their effect on stem cell viability.

**Figure 3:**
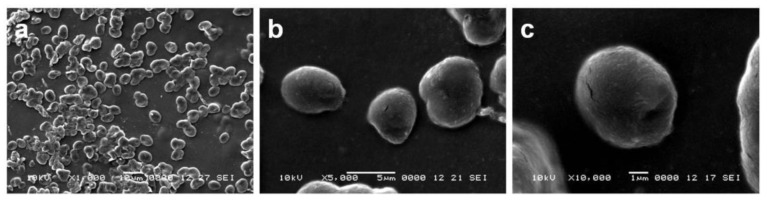
SEM images of multilayer BSA gel microparticles, magnification of 1000× (**a**), 5000× (**b**) and 10,000× (**c**).

The shape and size of the microcapsules were evaluated by SEM, after deposition of the suspension on a slide and drying at room temperature, Figure 3. The images show uniform size distribution for the microcapsules, with an average diameter of 5 μm. The morphology of the particle surface is smooth, as a qualitative indicator of the effectiveness of loading, as shown elsewhere [24].

**Figure 4:**
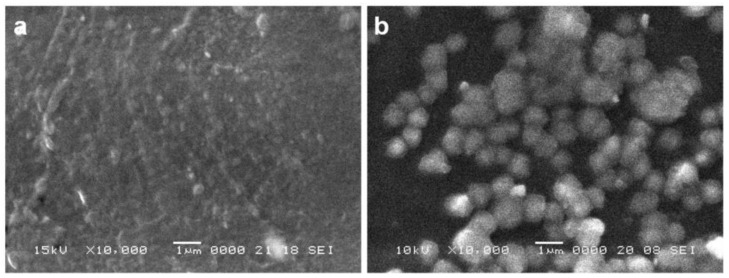
SEM images of a single chitosan hydrogel film (**a**) and of a chitosan hydrogel film containing the loaded microcarriers (**b**).

In order to check the way microcarriers are engulfed in the coating on top of titanium samples, a single chitosan hydrogel film was compared microscopically to the film containing microcapsules, Figure 4. Completely engulfed, flattened microcapsules, as resulted by drying during the process of obtaining the final coating film can be observed, oppositely to the relatively uniform morphology of the single chitosan hydrogel film.

### 3.2. Study of Growth Factors Release from the Titanium Implants-Chitosan Film-Microcapsules System

The quantitative assessment of the growth factors BMP2 and IGF1 released in the culture medium was performed with ELISA at days 2, 3, 7, 14, 17, and 21. The following distribution of wells was employed: (a) wells with only MSCs, as reference; (b) wells with uncoated titanium samples, as control; (c) wells with uncoated titanium samples, with GFs added in the culture medium; (d) wells with titanium samples coated with microcarriers releasing the studied GFs.

**Figure 5:**
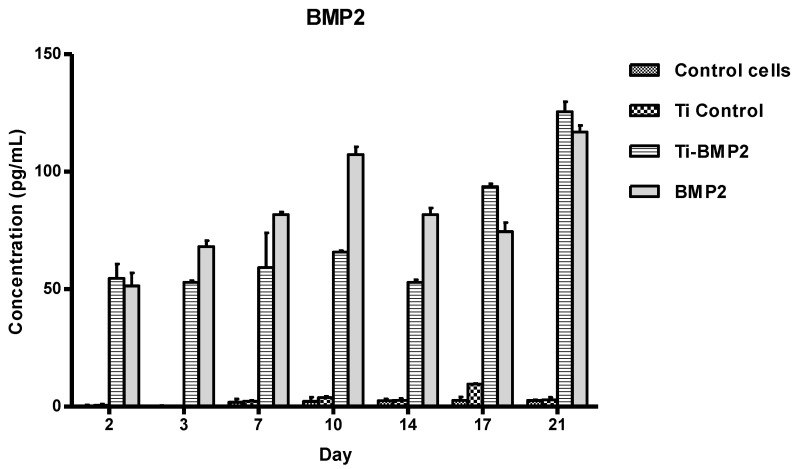
BMP2 concentrations at different time intervals in the culture medium samples from the wells with and without titanium implants. Legend: Control cells: cells seeded in wells containing only culture medium; Ti Control: uncoated titanium implants; Ti-BMP2: titanium implants coated with chitosan film containing microcapsules loaded with BMP2; BMP2: culture medium with added BMP2, without titanium implants.

The mean values of BMP2 (pg/mL), as measured spectrophotometrically in each well, are shown in Figure 5. For statistical analysis we used the software GraphPad Prism version 5 (GraphPad Software, San Diego, CA, USA). 2-way ANOVA and Bonferroni posttests show highly significant increase of BMP2 concentration in the wells treated with Ti-BMP2 and BMP2 versus controls (*p* < 0.001).

**Figure 6:**
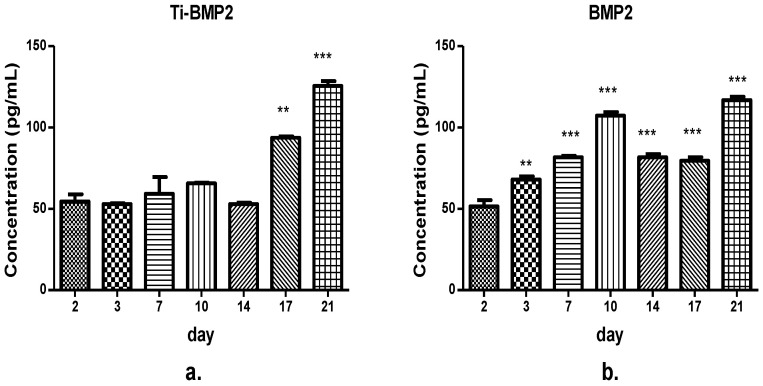
The concentration levels of bone morphogenetic protein 2 at different time intervals (days 2–21): (**a**) in the presence of titanium implants (Ti-BMP2) and (**b**) in the absence of titanium implants (BMP2) in the culture media. **—*p* < 0.001, ***—*p* < 0.0001 where *p* < 0.05 is considered the level of statistical significance.

The concentration levels of the growth factor BMP2 in the culture media from the wells with and without titanium implants are detailed in Figure 6. There was a time-dependent increase of BMP2 levels in both types of samples. This growth factor had a significant increase starting with day 7 in the culture media from the wells without implants (*p* < 0.0001), whilst in the samples from the wells containing titanium implants, there was a significant increase in days 17 and 21 (*p* < 0.0001) (one-way ANOVA).

The amount of BMP2 released from the titanium implants-chitosan film-microcapsules system was relatively constant in the first two weeks. The significant increase after this time could be the result of the differentiation of cells towards the bone lineage (Figure 6a). As for the directly introduced BMP2 in the culture medium, the high amounts detected during this time interval prove that it is less effectively captured by cells. In addition, it is increased at the end of the observation period is probably due to the terminal differentiation of stem cells (Figure 6b).

**Figure 7:**
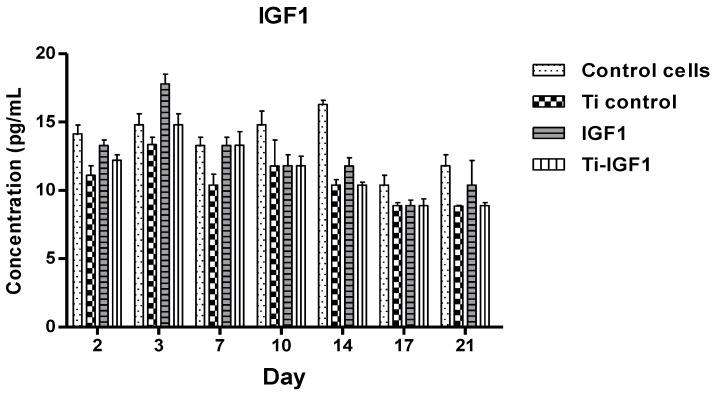
IGF1 concentrations at different time intervals in the culture medium samples from the wells with and without titanium implants. Legend: Control cells: cells seeded in wells containing only culture medium; Ti Control: uncoated titanium implants; Ti-IGF1: titanium implants coated with chitosan film containing microcapsules loaded with IGF1; IGF1: culture medium with added IGF1, without titanium implants.

The mean values of IGF1 (pg/mL), as measured spectrophotometrically in each well by the same ELISA technique, are shown in Figure 7. In all samples, high concentrations of this factor were seen on days 2 and 3, followed by a continue decrease until the end of the observation period.

**Figure 8:**
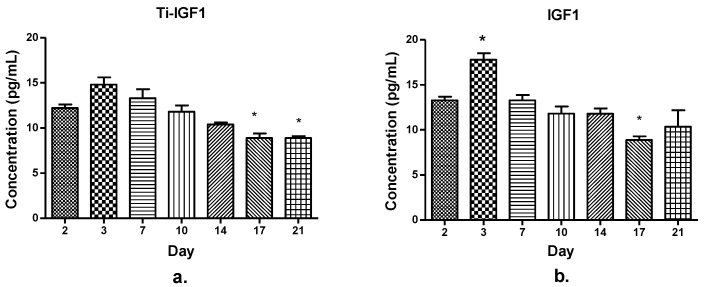
The concentration levels of insulin-like growth factor 1 at different time intervals (days 2–21) in the presence of titanium implants (**a**) and without titanium implants (**b**) in the culture media. *—*p* < 0.05, where *p* < 0.05 is considered the level of statistical significance.

The concentration levels of the growth factor IGF1 in the culture media from the wells with and without titanium implants are detailed in Figure 8.

An IGF1 concentration peak occurred in day 3 in the samples from wells containing titanium implants (Figure 8a) as well as in those containing cells and directly added IGF1 (Figure 8b). After that, there was a decrease in this GF’s amount, more significant at the end of time period (days 17 and 21). This behavior could be explained by IGF1 involvement, especially in the cellular proliferation processes, determining its time-dependent consumption as the cells proliferate.

### 3.3. BM MSCs Viability and Proliferation

PKH stain, which is a membrane linker and a viability marker, was used to assess cell viability at various time intervals. Cell adhesion and spreading are important early steps, which can give information about the biocompatibility of implants, as well as of subsequent cell proliferation. These cellular processes are related to surface properties (chemical composition, roughness, wettability).

**Figure 9:**
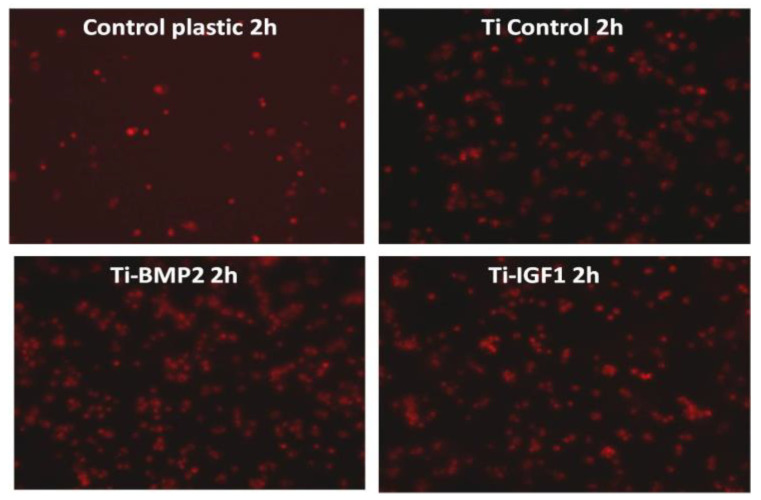
Cells with red fluorescence at the surface of the plastic wells and of the titanium discs with and without microcapsules containing the growth factors BMP2 and IGF1, at 2 h of cultivation (PKH staining).

The images captured at 2 h after PKH staining show an increased number of cells that tend to adhere to the surface of titanium implants with microcapsules loaded with growth factors, especially BMP2, as compared with the controls (Figure 9).

**Figure 10:**
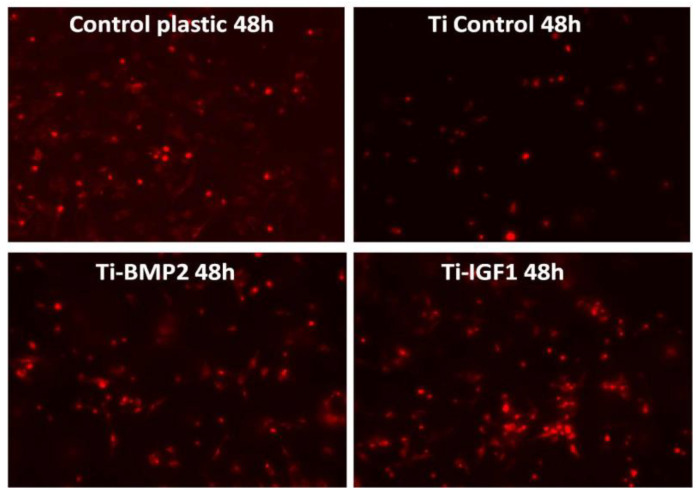
Cells with red fluorescence at the surface of the plastic wells and of the titanium discs with and without microcapsules containing the growth factors BMP2 and IGF1, at 48 h of cultivation (PKH staining).

After 48 h, the BM MSC cells started to spread on the surfaces. They are much more displayed and in an increased number on the titanium implants with growth factors, more pronounced for IGF1 (Figure 10).

**Figure 11:**
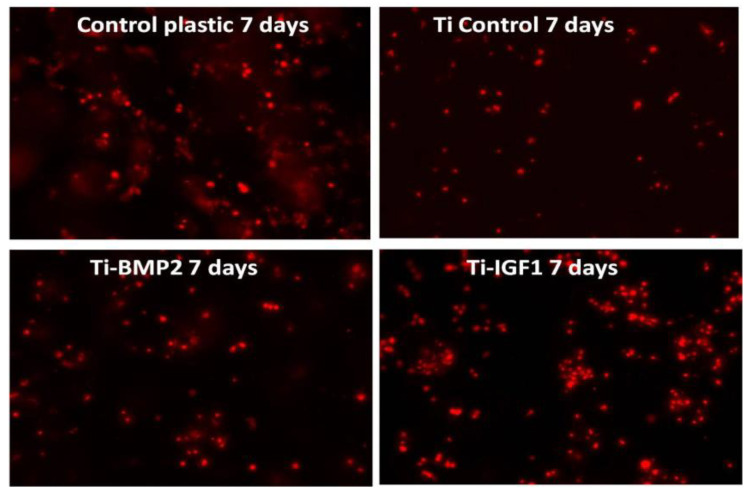
Cells with red fluorescence at the surface of the plastic wells and of the titanium discs with and without microcapsules containing the growth factors BMP2 and IGF1, after 7 days of cultivation (PKH staining).

The images captured after 7 days of cultivation show that the tendency observed at 48 h is maintained, with higher proliferation in samples containing IGF1 (Figure 11).

### 3.4. BM MSCs Osteogenic Differentiation on the Titanium Implants-Chitosan Film-Microcapsules System

The osteogenic differentiation of the stem cells under the influence of growth factors delivered by the titanium implants-chitosan film-microcapsules system was studied by performing immunocytochemical staining for the specific bone antigens osteopontin and osteocalcin, at 7 and 21 days of cultivation.

**Figure 12:**
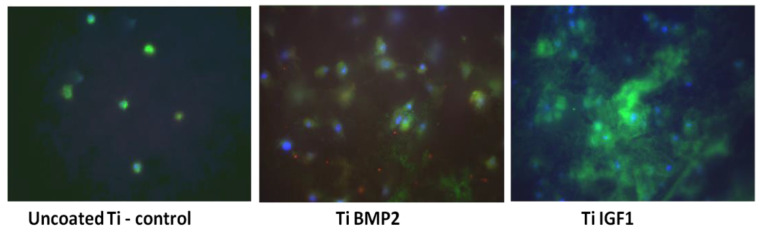
Immunocytochemical staining of BM MSC cells cultivated onto titanium implants for 7 days, with FITC (green) for osteopontin and phalloidin TRITC (red) for actin F. The actin F fibers could not be viewed in these captured images.

Evaluation at 7 days for osteopontin expression and rearrangement of actin F fibers (as an indicator of spreading) showed a more sustained proliferation rate for titanium implants with microcapsules loaded with growth factors, as compared with controls. The stronger expression of OP was on the titanium implants with IGF1. The cellular spreading was better observed on the implants with BMP2 (Figure 12).

**Figure 13:**
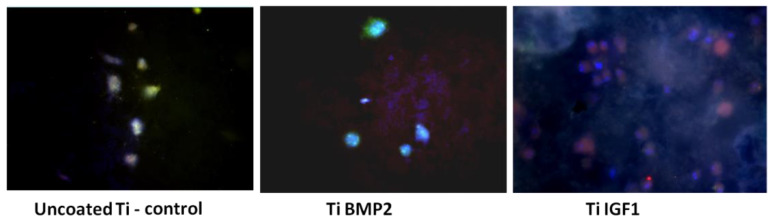
Immunocytochemical staining of BM MSC cells cultivated onto titanium implants for 21 days, with Texas red (red) for osteopontin OP and FITC (green) for osteocalcin OC.

After 21 days of cultivation, immunocytochemical staining for osteopontin and osteocalcin was performed, to assess both proliferation and differentiation. In this experiment, osteopontin was conjugated with Texas red and osteocalcin with FITC. The images in Figure 13 show less proliferation on titanium controls and titanium-BMP2 samples (stronger expression of OC marker as compared with OP). The implants with microcapsules loaded with IGF1 supported both cell growth and differentiation to the bone lineage, presenting positivity for both markers.

### 3.5. In Vivo Analysis

#### 3.5.1. Histology

**Figure 14:**
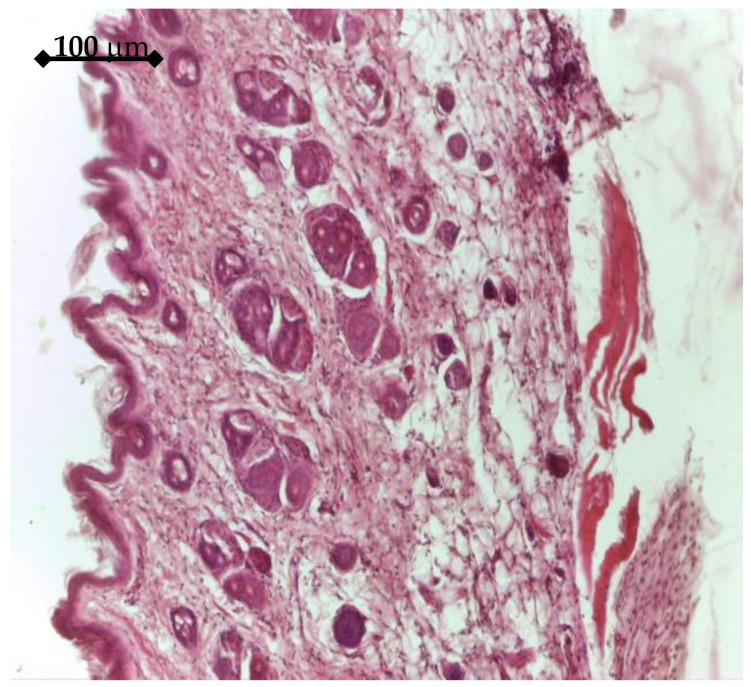
Tissue around Ti control sample (14 days), Hematoxylin eosin staining: tegument with thin epidermis, superficial and deep dermis, 10×.

**Figure 15:**
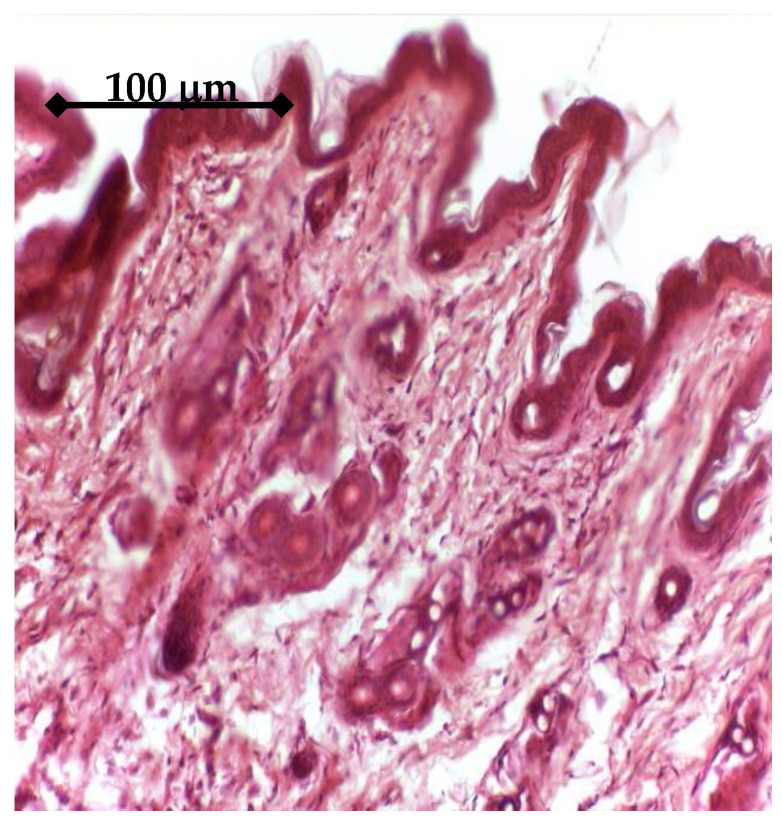
Tissue around IGF 1 Ti sample (14 days), Hematoxylin eosin staining: Superficial dermis with numerous bundles of collagen fibers, 20×.

In vivo experiments started with histology analysis, which was performed on tissue around GFs active or uncoated Ti samples. After 14 days, the tissue around uncoated Ti control explants show a normal structure, Figure 14. The IGF1 releasing samples determined a specific aspect of adjacent tissue after 14 days, Figure 15. A discrete fibrosis process could be observed both at the level of superficial dermis, with an increased number of collagen fibers, and at the level of deep dermis.

**Figure 16:**
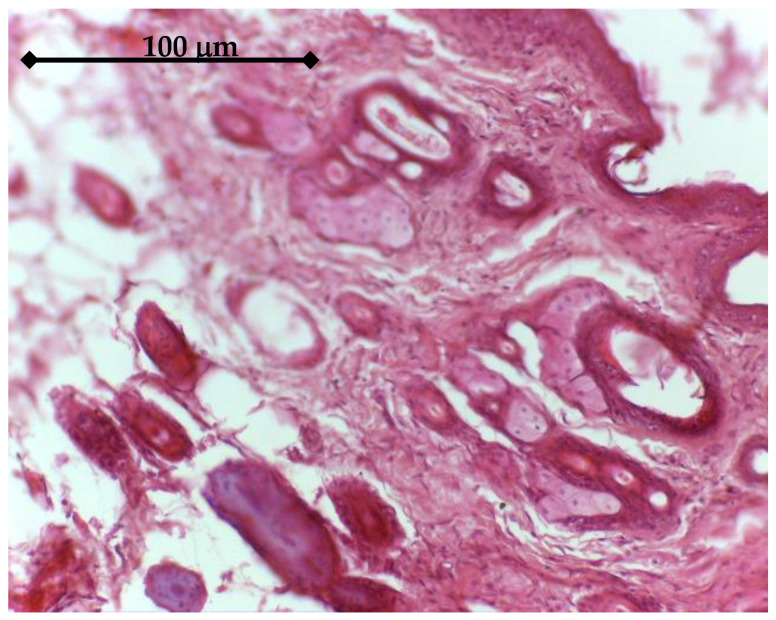
Tissue around BMP2 Ti sample (14 days), Hematoxylin eosin staining: Tegument with numerous bundles of collagen fibers, both in the superficial dermis and in the deep dermis, 20×.

BMP2 releasing samples had a different histologic effect. After 14 days, an accentuated fibrosis process could be seen in the dermis, both at the superficial level, where an increase in the number of collagen fibers could be observed, and at the deep level, Figure 16. In the vicinity of the implant, mesenchymal tissue, with cellular star-like elements can be seen.

#### 3.5.2. MicroCT

**Figure 17:**
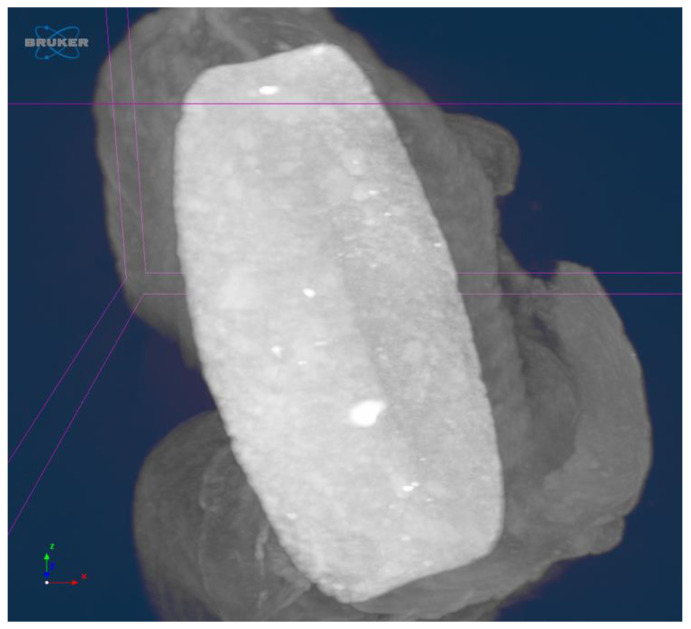
Tissue around IGF1 implant, 14 days, MicroCT image.

**Figure 18:**
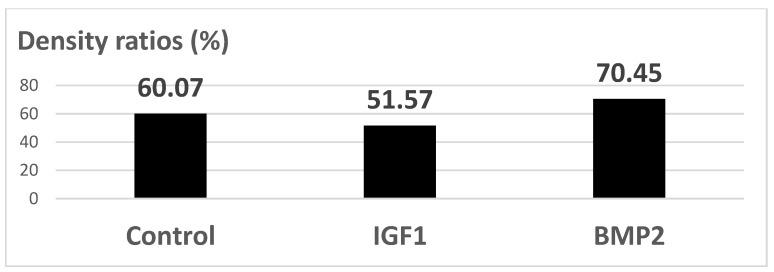
Relative density of tissue adjacent to samples vs. porous Ti density ratios [%], as seen by numerical analysis performed on MicroCT images.

The MicroCT images showed a marked texture for BMP2 releasing samples, with dense neoformation tissue in the vicinity of implants, while the uncoated Ti control and IGF1 releasing samples have similar aspects, Figure 17. Figure 18 shows the mean densities of tissue, as obtained by the numerical analysis, using the CTAn software (1.13.0.0, Skyscan, Bruker, Belgium), on MicroCT images. The results were obtained by comparing the density of tissue in direct contact to the samples (after virtually 3D rotating the image) measured in five distinct areas per sample (on the planar face of samples), and the as-resulted density corresponding to each titanium sample. This method was developed in order to avoid the lack of accuracy due to the peculiar conditions of each scanning. As it can be seen, BMP2 led to the highest densities, while IGF1 implants were surrounded by a less dense tissue compared to the uncoated Ti control ones.

Both the results of histological analysis and quantitative analysis of the density in the opacity profile using microCT showed that the growth factor BMP2 was the most effective in differentiating cells to bone tissues. In the histological analysis, the samples treated with BMP2 were the ones with the most marked fibrosis in both the superficial and the deep dermis. In addition to this accentuated fibrosis, a mesenchymal tissue and numerous neoformation vessels were observed in the vicinity of the implants. From the histological and microCT analyses, we can conclude that the GFs releasing porous Ti samples are a good support structure for stem cells and that they differentiate into bone tissue (through fibrous tissue).

## 4. Discussion

The proposed way of manufacturing the novel 3rd generation Ti-based multilayer was found to be effective from all points of view. The resulted structure of the substrate, with rounded pores of an appropriate size for a vital bone ingrowth and high open porosity, is optimal for endosseous implants designed for long skeletal bones. For this peculiar application, not only the size and shape of pores, required by the need to host functional osteons, but also the overall porosity, leading to a decrease in stiffness, is important. As reported elsewhere [24] using the same technological parameters, the Young’s modulus of porous titanium samples is around 6 GPa, highly appropriate for avoiding the mechanical mismatch. In addition, this comes with an ultimate tensile strength of about 600 MPa [24], enough for endosseous implants. Furthermore, the complex layer that coats the porous titanium specimens was found to be appropriate for the effective release of chemically stable growth factors, such as BMP2 and IGF1. Natural transporter polymers were evaluated in time for the delivery of GFs in bone tissue, due to their affinity towards proteins and their mild processing conditions [25]. Amongst the hydrogel generating polysaccharides for such use, chitosan is the only cationic one, due to glucosamine groups. Chitosan is poorly water soluble but, after the protonation of amino groups in glucosamine residues in diluted acids, it becomes a soluble polycation, able to form complexes on interaction with a wide variety of anionic species. Pectin is an edible water-soluble polysaccharide in many varieties, with uses as emulsifying agents, suspension stabilisers, muco-adhesive agents, and drug delivery systems [26]. The capacity of chitosan and pectin to form polyelectrolytic complexes through electrostatic interactions is widely exploited in the synthesis of nano/micro-transporters for specific colon drug delivery [27]. Bovine serum albumin (BSA), used in this work for the core of carrier particles, shows the advantage of being biocompatible, biodegradable, non-toxic, and non-immunogenic [28], thus of being largely used for the delivery of large or small hydrophobic drug molecules [29]. The microcarriers, representing the active constituent of the structure, were conceived as core-shells that, after loading, deliver the growth factors through the chitosan film they are engulfed in, in a stable and prolonged manner. Both the BSA gel core and the multiple double layers of calcium crosslinked pectin hydrogel/polyelectrolytic complex hydrogel pectin-chitosan assure, as demonstrated by ELISA method, a proper environment for an effective growth factors loading-releasing process. The coating method, very simple and reproductible, has led to a uniform distribution of the microcarriers conceived to deliver the growth factors. The overall effect of the microcarriers structure and chitosan film that engulfs these is a controlled release of growth factors during a long enough time period to observe significant induced biologic response both in in vitro and in vivo conditions. The 3rd generation Ti-based biomaterials released the growth factors in a more suitable manner compared to the tested direct in-well delivery method. Due to their effect in the early cellular processes, growth factors are considered for post-implantation local therapy, but the outcomes are affected largely by their fast degradation and appropriate delivery methods are under research [30]. One study’s method of using the effect of growth factors with titanium implants is to decorate these with a type of growth factors, such as human bone morphogenetic protein 7 (BMP7), by the means of an attachment layer, such as a sequence of poly(ethyl acrylate) (PEA) and fibronectin [31]. This system was proven, by the means of in vitro tests performed with human mesenchymal stem cells, to maximize osseointegration. Nevertheless, possible cytotoxic and inflammatory effects related to over-physiological doses, as well as the formation of ectopic bone after direct delivery of GFs, may occur [32]. Thus, a strategy to control the GFs delivery rate is required for an appropriate tissue effect. For instance, BMP2 is reported to be able to be encapsulated into micro/nanocarriers, leading to a controlled delivery rate via a controlled particle degradation rate [33]. More, subsequent entrapment into a hydrogel network was found to lengthen the biologic effect [34]. The same approach was employed in this work, the core-shell microcarriers engulfed in the chitosan coating proving reliable on-site delivery of the tested GFs.

According to their action mechanisms, the two growth factors were found to be captured by cells after a slowed-down release, which offers a very good way to assist cellular processes, as proven by viability, proliferation, and differentiation assays. It is pertinent to mention that, compared to direct delivery in the implanted site, leading to possible risks of GFs denaturation and adverse body reactions, the proposed route is safer and more convenient. The viability and proliferation of cells were significantly improved by the release of GFs from the proposed Ti-based multilayered materials, compared to the Ti uncoated samples obtained through the same powder metallurgy route. For the seven-day period, the most favorable in terms of cell proliferation but also differentiation to the bone lineage were the active Ti samples releasing growth factors, with an obvious advantage in terms of support of cell proliferation for IGF1. For 21 days, the released BMP2 did not support the proliferation of cells, leading to a process of terminal differentiation, while IGF1 supported both cell growth and differentiation to the bone lineage. To conclude, in vitro tests first showed the GFs loading in the microcapsules; then their release through the chitosan hydrogel film in the proposed system were found to be effective. The BM MSCs cells uptake-release kinetics during the 21 days interval was different for the two GFs and directly related to the cellular processes they influence. IGF1 and BMP2 were successfully delivered to cells by the proposed experimental system. In our experiments, the proposed delivery system of the factors involved in the bone growth and differentiation proved to be safe and convenient.

The in vitro results were sustained by in vivo findings, both as histology analysis and MicroCT imaging. As an overall result of the biologic evaluation of the effect of the studied GFs release, both in in vitro and in vivo conditions, one can see that IGF1 and BMP2 are complementary in terms of time scale and mechanisms of assisting the cellular processes. By prolonging the time for proliferation of cells and supporting their growth and differentiation, IGF1 is very helpful for post-implanting wound healing, while BMP2 is extremely useful for the earlier development of bone tissue. In addition, our experiments have shown that the incapsulated GFs were chemically stable in all cases. An interesting further development of this study may be the use of 3D bioprinting to depose the GFs delivering layer on top of the titanium specimens. A bio-ink [35] containing the loaded microcarriers builds the coating layer obtaining a bone-morphology geometry, which leads to better osseointegration. Next developments of our present research will focus on the delivery of both IGF1 and BMP2 growth factors together, with various released amounts, in view of optimizing a consistent 3rd generation biomaterial for bone implants designed to assist wound healing and vital bone growth.

## 5. Conclusions

The proposed multi-layered material consisting of a porous titanium substrate obtained via powder metallurgy route, coated with a chitosan film engulfing microsphere consisting of BSA gel bulk and double layers of calcium crosslinked pectin hydrogel/polyelectrolytic complex hydrogel pectin-chitosan, loaded with IGF1 and BMP2, acted as a proper 3rd generation biomaterial used for endosseous applications. The system was found to effectively release BMP2 and IGF1 growth factors post implanting, in a stable and active form that assures short and effective osseointegration and wound healing.

## Data Availability

The data presented in this study are available on request from the corresponding author. The data are not publicly available due to local regulations.
